# Analyzing Breathing Patterns in the Breaststroke Technique Through Dual-Media Kinematics and Fractal Dimension

**DOI:** 10.3390/s25103104

**Published:** 2025-05-14

**Authors:** Miriam Alves, Pedro Fonseca, Aléxia Fernandes, André V. Brito, Tiago M. Barbosa, João Paulo Vilas-Boas

**Affiliations:** 1 Centre of Research, Education, Innovation and Intervention in Sport (CIFI2D), 4200-450 Porto, Portugal; up201505002@edu.fade.up.pt (M.A.); up201402859@edu.fade.up.pt (A.F.); up201902341@edu.fade.up.pt (A.V.B.); 2 Porto Biomechanics Laboratory (LABIOMEP-UP), 4200-450 Porto, Portugal; pedro.labiomep@fade.up.pt; 3 Faculty of Sport, University of Porto, 4099-002 Porto, Portugal; 4 Department of Sport Sciences, Instituto Politécnico de Bragança, 5300-252 Bragança, Portugal; barbosa@ipb.pt; 5 Research Centre for Active Living and Wellbeing (LiveWell), Instituto Politécnico de Bragança, 5300-253 Bragança, Portugal

**Keywords:** swimming, breathing patterns, kinematics, center of mass

## Abstract

The most hydrodynamic swimming position occurs with the head submerged, highlighting the benefit of reduced breathing frequency for efficiency. This study aimed to characterize and compare kinematics between two breaststroke breathing patterns—breathing every cycle and breathing every two cycles—while also analyzing intra-cyclic velocity variation (*dv*) and fractal dimension. In the breathing every cycle pattern, each cycle included a breath. In the breathing every cycle pattern, swimmers breathed once per cycle. In the breathing every two cycles pattern, breathing occurred every second cycle, resulting in three types of cycles: breathing, non-breathing, and the breathing cycle following a non-breathing cycle. To ensure familiarity with the new breathing pattern, swimmers underwent a six-week intervention program. They then performed three maximal 25 m bouts in each breathing pattern. Kinematic data were collected using a dual-media optoelectronic system (Qualisys AB, Sweden), integrating underwater and dry-land camera recordings. The results showed minimal differences between the three cycle types. The non-breathing cycle had the shallowest and deepest head positions, the lowest horizontal head amplitude out of water, and the smallest vertical head amplitude. It also had the fastest maximum vertical velocity of the feet and maximum center of mass velocity in the swimming direction.

## 1. Introduction

Achieving high swimming velocity requires optimizing propulsion while minimizing resistance [1]. The most hydrodynamic position in swimming is achieved, in general, when the head is submerged, highlighting the importance of minimizing breathing frequency during a race to preserve efficiency [2]. Studies have shown that expert swimmers take fewer breaths to maintain an optimal streamlined body position and sustain efficient propulsion [3]. However, excessively minimizing breathing frequency may compromise propulsion, as breathing also supports rhythm and oxygen supply [2]. This balance is particularly influenced by race distance, with shorter events often allowing reduced breathing frequency, while longer races may require more frequent breathing to meet physiological demands [4]. Nevertheless, there is limited research on the impact of breathing on kinematics across various swimming techniques.

In butterfly technique, breathing has been shown to cause a decrease in velocity, increased body inclination during inhalation, and prolonged upper limbs recovery times [5,6,7]. Additionally, reduced propulsive continuity between upper and lower limb actions has been observed while breathing, resulting in a shorter downward leg kick (propulsive phase), extended less-propulsive phases (such as the upper limb catch and upward leg kick), and increased glide time [8]. In front crawl, breath-holding has been associated with faster 25 m time trials compared to higher breathing frequencies [4,8] and is a standard procedure undertaken by expert sprinters racing short distances. Breathing has been found to increase cycle duration and influence hand displacement patterns, resulting in shallower hand paths, slower vertical hand acceleration during the pull phase, and a slowdown in the horizontal velocity of the center of mass [9,10]. In breaststroke, where research is more limited, breathing every three cycles enabled swimmers to cover a 25 m distance in less time than breathing every cycle [11]. However, when comparing active drag and velocity between two breathing patterns, similar values were attained in 13 m breaststroke bouts [12].

Given that breathing changes body position and affects propulsion, it is paramount to understand how these changes influence velocity variation (*dv*), a key parameter in swimming performance [13]. Over the course of a swimming cycle, the intensity of hydrodynamic drag and propulsion vary constantly, since the motor actions of the upper and lower limbs and trunk are fairly discontinuous (especially in breaststroke and butterfly techniques). Thus, in each cycle, the velocity changes as a result of the positive and negative accelerations induced by the continuous variations of the resulting mechanical impulse [14]. Breaststroke has been defined as the swimming technique with the greatest *dv*, characterized by a two-peak profile [15]. A correlation between the *dv* and energy cost was reported [14,16], signaling that swimmers who exhibit fewer fluctuations are more likely to swim economically. Since breathing disrupts the swimmer’s streamlined position and causes fluctuations in propulsion, it is likely to influence *dv*. Understanding this relationship is essential, as *dv* reflects the extent of velocity fluctuations within a swimming cycle.

In the analysis of *dv*, discrete values are commonly used for quantification. Coefficient of variation computation is the mainstream procedure for quantifying *dv* [17,18,19,20] but the literature presents conflicting results, showing higher and/or lower *dv* in elite swimmers. For instance, see the following findings: swimmers achieve higher mean velocities when presenting lower intra-cycle velocity variation in the four swimming techniques [15]; higher mean velocities maintain their intra-cycle velocity variation values in the front crawl [21]; and no relationship was found between intra-cycle velocity variation and front crawl performance [22]. Some authors put forward that higher-level swimmers present lower intra-cycle velocity variation than lower-level or less experienced swimmers due to their improved technique, suggesting its inverse relationship with competitive level [19,23]. Conversely, others argued that elite swimmers display higher intra-cycle velocity variation due to higher cycle peak velocities and corresponding larger resistive forces [22]. Considering that swimming cycles have a time dimension, a continuous assessment of differences throughout the cycle would provide a complementary approach [7,24]. Therefore, using the Statistical Parametric Mapping could not only supplement the discrete *dv* data, but also offer a more detailed perspective on velocity variation over time.

Taking into consideration the state of-the-art research on *dv*, recent studies have incorporated nonlinear analyses like fractal dimension (*FD*) analyses to assess the complexity of motion in cyclic sports. Expanding on this approach, motor control methods have been applied to swimming to evaluate motion variation through stability and complexity calculations, providing additional insights into time–series variability [25]. *FD* is an invariant nonlinear parameter that characterizes systems exhibiting fractality or other properties that remain consistent over time and/or space [26]. As rule of thumb, as *FD* increases, so does the complexity of the time–series. Fractal dynamics in competitive sports have been reported in running, rowing, cycling, and swimming [25,27,28,29,30]. However, no study has examined whether changes in *FD* are sensitive to modifications in technical patterns, such as different breathing patterns in swimming.

Given the limited research on breaststroke breathing patterns and the impact of breathing on kinematics and nonlinear parameters, such as *FD*, in other swimming techniques, this study aimed to characterize and compare two breaststroke breathing patterns: breathing every cycle and breathing every two cycles. Additionally, we aimed to analyze and compare *dv* and *FD* between these two breathing patterns. In the breathing every cycle pattern, each cycle included a breath. In the breathing every two cycles pattern, each pair of cycles includes one non-breathing cycle followed by a breathing cycle, which we specifically refer to as the breathing cycle following a non-breathing cycle to avoid any confusion with the consecutive breathing cycles. This distinction leads to the study of three types of cycles: (i) the breathing cycle, (ii) the non-breathing cycle, and (iii) the breathing cycle following a non-breathing cycle. We hypothesized that the non-breathing cycle would be associated with changes in kinematics compared to the breathing cycle. Furthermore, we hypothesized that a reduced breathing pattern would lead to lower *dv* and reduced *FD*.

## 2. Methods

### 2.1. Participants

Fifteen swimmers (nine female swimmers and six male swimmers) from local swimming teams participated in the study. Their competitive level was determined based on their personal best performance in the 100 m breaststroke in a 25 m pool, assessed using FINA points as defined by World Aquatics. The primary physical and performance characteristics of the participants were as follows: 16.7 ± 5.5 and 15.9 ± 3.0 years old, 49.4 ± 10.1 and 57.0 ± 11.6 kg of body mass, 158.9 ± 9.2 and 169.3 ± 10.1 cm of height, and 355.7 ± 97.8 and 376.2 ± 122.6 FINA points for females and males, respectively. Swimmers were recruited based on the following criteria: (i) a minimum of five years of competitive swimming experience, (ii) participation at least at the regional competitive level, and (iii) absence of injuries in the six months prior to the evaluations. The study was approved by the local ethics committee (CEFADE 36/2022) and adhered to the principles outlined in the Declaration of Helsinki (2000). Participants (or their legal guardians, in the case of minors) were fully informed about the procedures, potential risks, and benefits of the study and provided written consent to participate.

### 2.2. Intervention Program

To familiarize swimmers with the pattern of breathing every two cycles, a six-week intervention program was implemented prior to the evaluation. The program consisted of 18 sessions (3 per week, 20 min each) conducted in a 25 m indoor pool. Designed to progressively increase difficulty, the program introduced challenges beyond the swimmers’ existing skill levels to facilitate skill acquisition [31]. The intervention began after the main competition of the season’s first macrocycle, coinciding with the transition to the second macrocycle. In the first session, swimmers watched a video demonstrating the new breathing pattern and were given the opportunity to self-explore it in the water. When the new breathing pattern was introduced, the swimmers were informed that the goal was to explore a new breathing pattern and to develop the technical ability to execute it consistently and effectively. The focus was on skill acquisition and familiarization rather than on performance enhancement per se. Swimmers were not told that this strategy was expected to improve or impair performance, in order to minimize potential expectancy effects. The aim was to ensure that participants approached the intervention with an open and exploratory mindset. During the first week, the coach provided feedback that gradually transitioned from analytical to holistic technical training. Over the next two weeks, swimmers completed three low-intensity sessions with increasing volume. In weeks four and five, the program emphasized increased intensity. The final week alternated between volume and intensity. Throughout the sessions, swimmers received targeted coaching feedback on technical aspects to enhance their performance.

### 2.3. Motion Capture System

Kinematics were collected using a dual-media optoelectronic system (Qualisys AB, Göteborg, Sweden), which merged the information recorded by an underwater and dry-land camera system. The underwater system included 7 cameras (6 × Oqus 300+u, 1 × Oqus 700+u, Göteborg, Sweden), while the dry-land system included 9 cameras (6 × Oqus 400, 3 × Oqus 310+). Both systems were synchronized and recorded at a 100 Hz sampling frequency.

The two systems were independently calibrated, starting with the positioning of a static L-frame to set the origin of their global coordinate system (GCS). Then, a dynamic calibration was performed with a fixed-length wand to obtain a calibrated volume of approximately 5.5 × 2.0 × 1.5 m in the middle of the pool. Next, a twin calibration was performed to align and superimpose the dry-land system GCS to that of the underwater system, which became the twin GCS. With this, a three-dimensional dual-media working volume was enabled, where the orthogonal axes were defined as Y for horizontal (swimming direction), X for mediolateral, and Z for vertical displacements, respectively. These procedures were performed in accordance with the manufacturer’s instructions (Qualisys Track Manager User Manual), resulting in a twin system error of less than 1.5 mm. To assess the water level relative to the twin system GCS origin, a short data acquisition method was carried out. For that purpose, the calibration wand was placed parallel to the water surface, at its height. Thereafter, all values in the Z-axis were considered negative if performed below this level.

**Figure 1:**
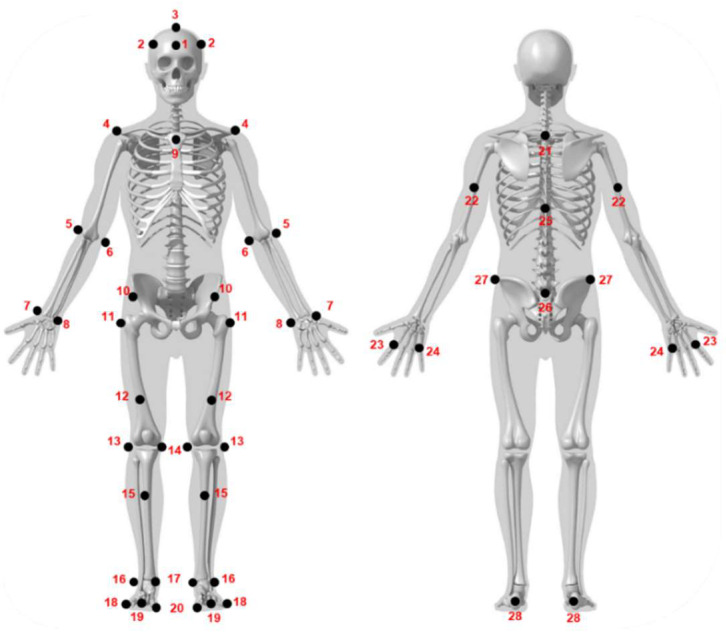
Schematic representation of the full-body swimming marker set.

In order to model the swimmers throughout the tests, a set of 50 retroreflective markers were placed on pre-selected anatomical landmarks. These were based on a modified version of the Qualisys Sports Marker Set (2021) [32], with the addition of the following markers: #3 on the head, #24 on the hands, and #16 and #20 on the feet (Figure 1, Appendix A.1). Each spherical marker had a diameter of 19 mm and was attached to a 20 mm flat base. This base was first attached to the skin with double-sided tape and was then covered with a transparent waterproof tape (Euroderm, Eurofarm, Italy). To avoid peeling due to water infiltration, the edges of this tape were reinforced with kinesiology tape (Leukotape, BSN medical), similar to the waterproofing procedure reported in electromyography [33]. Once this process was finished, the marker was attached to the base.

### 2.4. Data Collection Procedures

The evaluation sessions were conducted in a 25 m indoor pool with a depth of 1.80 m. Swimmers completed a typical 1000 m individual race preparation warm-up. Afterwards, they performed three maximal bouts of 25 m in each breathing pattern—breathing every cycle and breathing every two cycles—with the order of the breathing conditions randomized to minimize order effects. No differences were found between the time trials, indicating that there was no observable order effect on performance. Between trials, swimmers passively rested for four minutes, a period considered sufficient to avoid the acute and cumulative effects of fatigue [34]. After each trial, all swimmers were informed of their trial time, which was expected to be within ±2.5% of the targeted race pace. If this was not achieved, the subject was invited to repeat the trial. This approach was chosen to minimize the number of repeated trials due to incorrect pacing or deviation from the intended maximal effort, which could also compromise data quality and induce fatigue. Out of the 90 bouts performed, 11 did not meet the predefined criteria and were repeated.

### 2.5. Data Analysis and Processing

The markers’ trajectories were identified in Qualisys Track Manager (Qualisys AB, Sweden), and trajectory gaps were filled with polynomial and relational interpolations. Then, the resulting trajectories were exported to Visual 3D v 5.01.21 (HAS-Motion, Ontario, Canada), where they were low-pass-filtered with a 6 Hz Butterworth low-pass filter [35], and a 6 degrees-of-freedom biomechanical model was created. The same filter was applied to the calculated joint angles.

A full cycle was set as the period from the beginning of the upper limbs’ outsweep until the same discrete event happened again. Two consecutive swimming cycles, taken in the middle of the calibrated volume, were analyzed. A total of 173 cycles were studied. Three types of cycles were defined: (i) the breathing cycle, (ii) the non-breathing cycle, and (iii) the breathing cycle following a non-breathing cycle. This means that, in the breathing every cycle condition, all cycles were breathing cycles. In contrast, in the breathing every two cycles condition, each pair of cycles consisted of one non-breathing cycle followed by a breathing cycle—which we specifically refer to as the “breathing cycle following a non-breathing cycle”. This distinction allowed us to analyze how the presence or absence of a preceding breath or non-breathing cycle influenced kinematics.

The analyzed variables were categorized as follows: (i) movement amplitude, (ii) joint angles, and (iii) swimming velocity. The movement amplitude described the motor path of the center of mass of the hands, feet, head, and the body’s CoM during a complete swimming cycle. The centers of mass of each body segment and the full body were automatically calculated by the software (Visual 3D v 5.01.21), according to anthropometrical biomechanical tabled values (Hanavan anthropometrical model). Vertical movements included minimum and maximum depths, as well as their amplitudes (the differences between these values). Horizontal amplitude measured the displacement between the most forward and rearward points, while medial–lateral amplitude accounted for the lateral variation in position. Two indices complemented these measurements [36]: the horizontality index (ratio between medio-lateral and vertical amplitudes during the propulsive phases) and the anterior–posterior stabilization index in the sagittal plane (ratio between vertical and horizontal amplitudes during the propulsive phases). Joint angles assessed the hip, knee, and trunk sagittal amplitude. Finally, velocities were analyzed for the CoM, feet, hands, and head, considering minimum, mean, and maximum values in each direction (X, Y, and Z axes). For variables of the feet and hands, the average between left and right side were considered. All these variables are further described in detail in Appendix A.2.

The intra-cycle mean variation and the fractal dimension were derived from the CoM velocity in the swimming direction. Intra-cycle mean velocity variation was obtained by the calculation of the coefficient of variation, as suggested by [20] and reported in the literature [17,18,19]. Fractal dimension (*FD*) was determined using Higuchi’s method [37] which has also been applied in swimming research [25,38]:

$$FD=\frac{d\;\log\;N\;(L\;\left(k\right))}{d\;\log\;(k)}$$

where *FD* is the fractal dimension, *N* is the number of new points from the speed–time–series data, and *k* is the scaling factor.

### 2.6. Statistical Analysis

Mean, standard deviation, and 95% confidence intervals were calculated for all variables. Data normality was assessed using the Shapiro–Wilk test. A repeated-measures ANOVA (within-subjects) was conducted to compare the three cycle types: breathing cycle, non-breathing cycle, and breathing cycle following a non-breathing cycle. Statistical significance was set at *p* < 0.05. Then, post hoc tests with Bonferroni correction were performed. Effect sizes were evaluated using partial eta squared (*η_p_*^2^) and interpreted as follows: no effect (0 < *η*^2^ ≤ 0.04), small effect (0.04 < *η*^2^ ≤ 0.25), moderate effect (0.25 < *η*^2^ ≤ 0.64), and strong effect (*η*^2^ > 0.64) [39]. To further analyze velocity fluctuations throughout the stroke cycle, breaststroke cycles were time-normalized to 101 data points, and Statistical Parametric Mapping (SPM) ANOVA tests were applied using MATLAB R2023b (The MathWorks Inc., Natick, MA, USA).

## 3. Results

The data revealed that the minimum and maximum head depths varied across the three cycles. In Table 1, both the breathing cycle and the breathing cycle following a non-breathing cycle showed deeper minimum depths (*p* < 0.001; *F* = 25.096; *ŋ*^2^ = 0.596) and shallower maximum depths (*p* < 0.001; *F* = 7.925; *ŋ*^2^ = 0.318) compared to the non-breathing cycle. Additionally, the head’s vertical amplitude was lower in the non-breathing cycle (*p* = 0.03; *F* = 8.919; *ŋ*^2^ = 0.344). The horizontal amplitude of the head’s motor path out of the water differed between the cycles (*p* < 0.001; *F* = 32.378; *ŋ*^2^ = 0.656), with the breathing cycle exhibiting the highest values, followed by the breathing cycle after a non-breathing cycle, and then the non-breathing cycle. In Table 2, the minimum medio-lateral velocity (X-axis) of the CoM also differed (*p =* 0.04; *F* = 6.094; *ŋ*^2^ = 0.264), with the breathing cycle showing the lowest value. Furthermore, the maximum vertical velocity (Z-axis) of the feet was higher in the non-breathing cycle compared to the other two cycles (*p* = 0.018; *F* = 4.482; *ŋ*^2^ = 0.209). The maximum velocity in the swimming direction of the CoM (Y-axis) was greater in the non-breathing cycle compared to other two cycles (*p* = 0.02; *F* = 7.180; *ŋ*^2^ = 0.297).

The breathing cycle and the non-breathing one differed in the medio-lateral mean velocity of the head (*p* = 0.029; *F* = 3.726; *ŋ*^2^ = 0.180). The breathing cycle and the breathing cycle following a non-breathing cycle differed in the medio-lateral amplitude of the hands (*p* = 0.08; *F* = 5.667; *ŋ*^2^ = 0.250) and in the minimum vertical velocity of the CoM (*p* = 0.005; *F* = 5.931; *ŋ*^2^ = 0.259). The breathing cycle presented higher values in the two variables.

Between the non-breathing cycle and the breathing cycle following a non-breathing cycle, several kinematic differences were observed. The mean vertical velocity of the feet was higher in the non-breathing cycle than in the breathing cycle following a non-breathing cycle (*p* = 0.010; *F* = 5.496; *ŋ*^2^ = 0.244). In contrast, the maximum vertical velocity of the head was greater in the breathing cycle following a non-breathing cycle than in the non-breathing cycle (*p* = 0.035; *F* = 3.830; *ŋ*^2^ = 0.184). Similarly, the mean velocity of the head in the swimming direction was higher in the non-breathing cycle than in the breathing cycle following a non-breathing cycle (*p =* 0.019; *F* = 4.101; *ŋ*^2^ = 0.194). Lastly, the minimum vertical velocity of the head was different between the non-breathing cycle and the breathing cycle following a non-breathing cycle (*p* = 0.039; *F* = 3.507; *ŋ*^2^ = 0.171).

**Figure 2:**
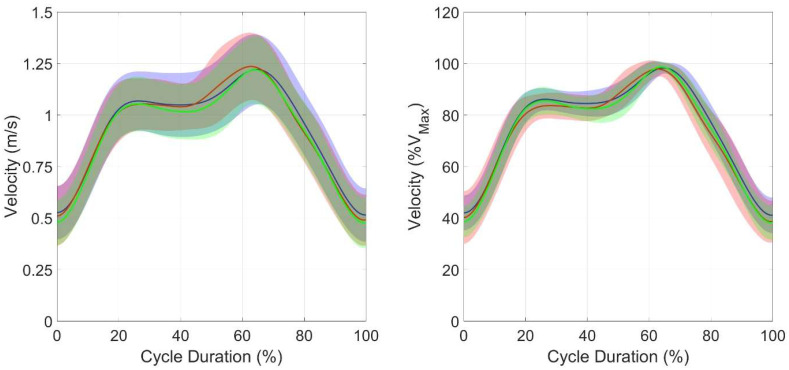
Mean group curves ± SD and SPM ANOVA test results. In the left panel, velocity values are presented in absolute values, while in the right panel, they are normalized as a percentage of the cycle maximum velocity. Blue: breathing cycle; red: non-breathing cycle; green: breathing cycle following a non-breathing cycle.

**Figure 3:**
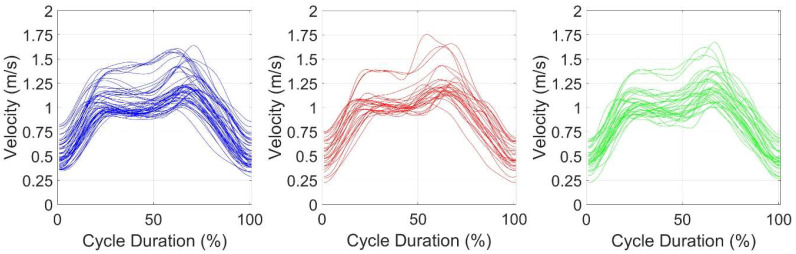
Overlapping representation of all recorded breathing cycles. Blue: breathing cycle; red: non-breathing cycle; green: breathing cycle following a non-breathing cycle.

**Figure 4:**
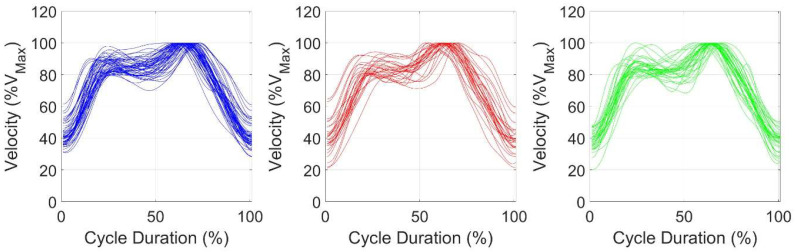
Overlapping representation of all recorded cycles. Velocity was normalized as a percentage of the cycle’s maximum values. Blue: breathing cycle; red: non-breathing cycle; green: breathing cycle following a non-breathing cycle.

No differences were found between the three cycles in the joint angles (Table 3), in the coefficient of variation in the *dv*, and in the *FD* (Table 4). In the SPM analysis (Figure 2, Figure 3 and Figure 4), the mean velocity curve was similar between the three cycles, demonstrating a comparable variation, and highlighting that the cycles are performed similarly. 

## 4. Discussion

The current study characterized and compared two breaststroke breathing patterns: breathing every cycle and breathing every two cycles. Additionally, we aimed to analyze and compare *dv* and *FD* between these two breathing patterns. We hypothesized that the non-breathing cycle would be associated with changes in kinematics compared to the breathing cycle. Furthermore, we hypothesized that a reduced breathing pattern would lead to lower *dv* and reduced *FD*. Our main results showed that the non-breathing cycle had both the minimum and the highest maximum head depths, the lowest horizontal amplitude of the head’s motor path out of the water, and the smallest vertical head amplitude. This cycle also presented the highest maximum vertical velocity of the feet which may have led to also presenting the highest maximum CoM velocity in the swimming direction compared to the other two cycle types. Lastly, the breathing cycle showed greater medio-lateral hand amplitude and a lower minimum CoM velocity than the breathing cycle following a non-breathing cycle.

This is the first study to examine the kinematic influence of different breathing patterns in breaststroke. Our findings indicate that the breathing cycle and the breathing cycle following a non-breathing cycle exhibited higher minimum and lower maximum head depths than the non-breathing cycle. This means that, although the non-breathing cycle showed head positions closest to the water surface level when the head was out of the water, it also exhibited the deepest positions when submerged. This was expected, as the swimmers did not lift their heads out of the water to breathe. However, it appears that not breathing causes the head to sink more during a swimming cycle than when swimmers lift their heads for breathing. The non-breathing cycle also showed the lowest vertical head amplitude, along with the lowest horizontal amplitude of the head above the water. This aligns with expectations, as in the non-breathing cycle, swimmers only break the water’s surface minimally to avoid disqualification. According to World Aquatics rules, swimmers are required to break the surface with their head, but it is not mandatory to breathe during this phase.

When swimmers breathe, their head rises slightly above the water surface, potentially increasing vertical displacement compared to the non-breathing cycle, where the head remains closer to the water level for longer periods. Lifting the head for breathing could also increase hydrodynamic drag by increasing the cross-sectional frontal area [40]. A less streamlined position may lead not only to increased drag but also to a higher energy cost [41,42,43]. However, the thorax angle in our study did not differ between the three types of cycles, and no changes in active drag between two breathing patterns in breaststroke were found in 13 m maximal bouts [12]. This may be due to the fact that the breaststroke technique has become flatter over the years compared to 20–40 years ago; therefore, the action of not breathing does not affect the trunk inclination. For instance, previous studies reported angles of 53° and 63° for more undulating styles [44,45], while this study observed angles of 45° ± 12°, which is consistent with more recent research [8,46].

The non-breathing cycle also showed a higher mean head velocity in the swimming direction compared to the breathing cycle following a non-breathing cycle. This may be because not lifting the head reduces vertical displacement, allowing swimmers to maintain higher forward velocity. By avoiding the interruption caused by head elevation for breathing, the swimmer may sustain a more continuous propulsion phase, which could contribute to maintaining velocity.

The non-breathing cycle exhibited the highest maximum CoM velocity in the swimming direction, and a higher maximum vertical velocity of the feet, suggesting that not breathing may enhance feet propulsion and, consequently, peak CoM velocity. However, despite achieving higher peak velocities, the overall mean and minimum velocities of the CoM were unaffected. One possible explanation for this is that, while the swimmer achieves higher velocities during the non-breathing phase, the rest of the cycle brings the velocity back to levels similar to those of the other cycles. This is further supported by the absence of differences in mean CoM velocity in the swimming direction in both the SPM analysis and *dv*.

The medio-lateral hand amplitude was greater in the breathing cycle, along with a lower minimum vertical velocity of the CoM, compared to the breathing cycle following a non-breathing cycle. This suggests that swimmers in the breathing cycle achieve a larger medio-lateral hand amplitude, which could contribute to increased propulsion. Additionally, it indicates that not breathing in the previous cycle may influence the subsequent breathing cycle by reducing medio-lateral hand amplitude, potentially diminishing propulsion. The lower minimum vertical velocity of the CoM in the breathing cycle suggests that this cycle may experience a greater velocity drop, potentially due to the increased drag caused by the elevated head position during breathing. This effect, though, did not reflect in the *dv*, which remained similar across cycles.

Intracycle velocity variation is a biomechanical variable that reflects the velocity fluctuation within a swimming cycle [15,47]. However, the literature presents conflicting results, reporting both higher and lower *dv* values in elite swimmers. One study concluded that the coefficient of variation was the only approach that was sensitive to both mean swimming velocity and instantaneous velocity dispersion within the cycle [48]. However, as highlighted by [49], the coefficient of variation should not be directly compared without accounting for the mean velocity, as differences in mean velocity can influence *dv* comparisons and lead to misinterpretations. When using the coefficient of variation as an index of *dv*, researchers and practitioners should always report and interpret the coefficient of variation alongside the mean and standard deviations to ensure accurate conclusions and meaningful feedback.

In breaststroke, previous studies have reported *dv* values of 32% for male breaststrokers [46] and 44.2 ± 2.4% and 48.5 ± 2.5% for elite and national-level breaststroke swimmers, respectively [7]. In our study, values ranged between 1.01% and 27.9%, with no differences between the three breathing cycle types. These relatively low coefficient of variation values may be attributed to the fact that the sample in this study includes young swimmers with a relatively low competitive level. As a result, they may lack the ability to generate significant accelerations, which could lead to lower *dv*. Furthermore, the three cycle types exhibited similar velocity variation, suggesting that the breathing pattern did not influence *dv*.

Recent studies have incorporated nonlinear analyses, such as *FD*, to assess motion complexity in cyclic sports. Altogether, higher *FD* values indicate greater complexity in the time–series [26]. In our study, *FD* was calculated with the Higuchi’s method, as it seems to be the most suitable for research involving time–series [50]. Reported *FD* values in front crawl range from approximately 1.84 to 1.95 [25,38], with values of 1.94–1.95 observed before and after a front crawl bout. A broader analysis across all four swimming techniques found *FD* values between 1.82 and 1.92, with breaststroke showing the highest complexity (*FD* = 1.92) [38]. Our *FD* values ranged from 1.11 to 1.55, with no differences between the three cycle types, suggesting similar complexity among them. These lower values compared to the literature may be attributed to the high variability within the sample, which could stem from differences in skill level and technique across the recruited swimmers. In fact, highly qualified, expert, and non-expert swimmers exhibited *FD* values of 1.84 ± 0.08, 1.85 ± 0.09, and 1.89 ± 0.06, respectively, showing that the level of expertise affects the complexity of the motor system [51]. 

Regarding the SPM analysis, the mean velocity curves were similar across the three types of cycles, highlighting similar patterns. This suggests that, despite some kinematic differences, the overall cycle remains consistent and, as seen above, has much lower complexity than expected. By observing the overlapping representation of all recorded breathing cycles, it becomes clear that swimmer’s ability to swim faster has a significant impact on the results, with lower-velocity and higher-velocity cycle differences becoming visible (Figure 3). By normalizing each cycle to its maximum velocity, this effect was tackled. Nevertheless, this also highlights the finding that swimmers reached their maximum velocity at different moments in time, leading to an overlapping effect at the top of the graph similar to a horizontal line. Indeed, the time of occurrence of the maximum and minimum velocities within the swimming cycle were not studied. However, this might be relevant, as it will relate to the swimming technique and the body position.

## 5. Limitations and Future Research

This study focused on three specific cycle types, limiting the scope of analysis to these predefined breathing patterns. Future research should explore additional breathing patterns, such as breathing every three cycles. Another key limitation was the variability within the sample, which may have influenced the results. A more homogeneous sample, such as elite swimmers with similar technical proficiency, could provide more consistent insights into the effects of such a detailed aspect of technique, such as different breathing patterns. Additionally, the sample size did not allow for the investigation of potential sex-related differences in the response to breathing strategies, which should be addressed in future studies. Furthermore, breathing pattern strategies over longer distances were not addressed in this study, as performance was analyzed at the cycle level rather than across the full 25 m trials or other distances. Future studies should investigate the impact of breathing frequency across longer race distances.

## 6. Conclusions

This is the first study on the influence of different breathing patterns in the breaststroke technique on the kinematics and nonlinear complexity of the swimmer’s movement. Differences between the three breaststroke swimming modes were minimal, and did not clearly point to the superiority of one of the breathing techniques over the others. Nevertheless, when not breathing, the swimmers kept their heads closer to the water level, and they exhibited the lowest horizontal head amplitude and the lowest vertical amplitude of the overall movement of the head when their head was out of the water. Additionally, this cycle presented the highest maximum vertical velocity of the feet and the highest maximal CoM velocity in the swimming direction; these data may suggest a relative biomechanical superiority. Finally, the breathing cycle showed greater medio-lateral hand amplitude and a lower minimum CoM horizontal velocity than the breathing cycle following a non-breathing cycle.

## Figures and Tables

**Table 1:** Movement amplitudes of the hand, foot, head, and CoM, along with repeated-measures ANOVA results.

	Variables	Breathing Cycle	Non-Breathing Cycle	Breathing Cycle Following a Non-Breathing Cycle	F	ŋ^2^
		Mean ± SD [95%Cl]	Mean ± SD [95%Cl]	Mean ± SD [95%Cl]		
Hands motor path	Min depth (m)	−0.02 ± 0.05	−0.03 ± 0.05	−0.03 ± 0.09	0.435	0.025
		[−0.01 ± −0.03]	[−0.01 ± −0.04]	[0 ± −0.06]		
	Max depth (m)	−0.34 ± 0.05	−0.35 ± 0.05	−0.32 ± 0.08	2.124	0.111
		[−0.33 ± −0.35]	[−0.34 ± −0.37]	[−0.3 ± −0.35]		
	Vertical amplitude (m)	0.32 ± 0.08	0.33 ± 0.08	0.29 ± 0.15	1.577	0.085
		[0.34; 0.31]	[0.35; 0.30]	[0.34; 0.25]		
	Horizontal amplitude (m)	1.11 ± 0.23	1.13 ± 0.24	1.11 ± 0.21	0.319	0.018
		[1.16; 1.06]	[1.20; 1.06]	[1.17; 1.04]		
	Medio-lateral amplitude (m)	**0.40 ± 0.06 ^c^**	0.40 ± 0.06	**0.38 ± 0.06 ^a^**	5.667	0.250
		[0.41; 0.39]	[0.42; 0.38]	[0.40; 0.37]		
	Horizontality index	1.46 ± 0.32	1.38 ± 0.37	1.39 ± 0.30	0.0078	0.205
		[1.53; 1.39]	[1.49; 1.27]	[1.49; 1.29]		
	Antero-posterior stabilization index in the sagittal plane	1.03 ± 0.76	0.90 ± 0.48	1.03 ± 1.10	0.2379	0.004
		[1.18; 0.87]	[1.04; 0.76]	[1.39; 0.68]		
Feet motor path	Min depth (m)	−0.15 ± 0.04	−0.13 ± 0.04	−0.13 ± 0.03	0.594	0.034
		[−0.14 ± −0.16]	[−0.12 ± −0.14]	[−0.12 ± −0.14]		
	Max depth (m)	−0.43 ± 0.07	−0.43 ± 0.06	−0.42 ± 0.06	1.840	0.098
		[−0.42 ± −0.45]	[−0.42 ± −0.45]	[−0.41 ± −0.44]		
	Vertical amplitude (m)	0.28 ± 0.04	0.30 ± 0.05	0.29 ± 0.04	1.842	0.100
		[0.29; 0.27]	[0.32; 0.29]	[0.30; 0.28]		
	Horizontal amplitude (m)	1.20 ± 0.21	1.16 ± 0.22	1.12 ± 0.23	1.603	0.086
		[1.24; 1.16]	[1.22; 1.10]	[1.20; 1.05]		
	Medio-lateral amplitude (m)	0.35 ± 0.05	0.35 ± 0.07	0.35 ± 0.05	0.831	0.047
		[0.36; 0.34]	[0.37; 0.33]	[0.37; 0.34]		
	Horizontality index	1.09 ± 0.25	1.10 ± 0.27	1.10 ± 0.22	3.722	0.057
		[1.14; 1.04]	[1.17; 1.02]	[1.17; 1.03]		
	Antero-posterior stabilization index in the sagittal plane	8.76 ± 22.70	9.60 ± 22.29	9.82 ± 13.24	2.890	0.045
		[13.40; 4.12]	[16.11; 3.08]	[14.15; 5.50]		
Head motor path	Min depth (m)	**0.26 ± 0.04 ^b^** **^,^** **^c^**	**0.14 ± 0.08 ^a^** **^,^** **^c^**	**0.26 ± 0.04 ^a^** **^,^** **^b^**	25.096	0.596
		[0.27 ± 0.25]	[0.16 ± 0.12]	[0.27 ± 0.25]		
	Max depth (m)	**−0.15 ± 0.06 ^b^**	**−0.18 ± 0.05 ^a^** **^,^** **^c^**	**−0.15 ± 0.05 ^b^**	7.925	0.318
		[−0.13 ± −0.16]	[−0.16 ± −0.19]	[−0.13 ± −0.16]		
	Vertical amplitude (m)	**0.40 ± 0.09 ^b^**	**0.32 ± 0.10 ^a^** **^,^** **^c^**	**0.41 ± 0.07 ^b^**	8.919	0.344
		[0.42; 0.39]	[0.35; 0.29]	[0.43; 0.39]		
	Horizontal amplitude (m)	1.13 ± 0.22	1.15 ± 0.22	1.11 ± 0.20	0.963	0.054
		[1.17; 1.08]	[1.22; 1.09]	[1.17; 1.04]		
	Horizontal amplitude of the motor path of the head out of the water (m)	**0.63 ± 0.08 ^b^**	**0.45 ± 0.10 ^a^** **^,^** **^c^**	**0.62 ± 0.08 ^b^**	32.378	0.656
		[0.65; 0.62]	[0.48; 0.42]	[0.65; 0.60]		
CoM motor path	Min depth (m)	−0.13 ± 0.02	−0.13 ± 0.05	−0.11 ± 0.07	0.672	0.038
		[−0.12 ± −0.13]	[−0.11 ± −0.14]	[−0.09 ± −0.13]		
	Max depth (m)	−0.23 ± 0.03	−0.23 ± 0.03	−0.23 ± 0.03	1.674	0.090
		[−0.23 ± −0.24]	[−0.23 ± −0.24]	[−0.22 ± −0.24]		
	Vertical amplitude (m)	0.11 ± 0.02	0.11 ± 0.04	0.13 ± 0.06	3.559	0.173
		[0.11; 0.10]	[0.12; 0.09]	[0.14; 0.11]		
	Horizontal amplitude (m)	1.14 ± 0.20	1.15 ± 0.21	1.11 ± 0.19	0.617	0.035
		[1.18; 1.10]	[1.21; 1.08]	[1.17; 1.05]		

CoM—center of mass; v—velocity; ^a^—differs from the breathing cycle; ^b^—differs from the non-breathing cycle; ^c^—differs from the breathing cycle following a non-breathing cycle.

**Table 2:** Three-dimensional velocities of the CoM, feet, hands, and head, considering maximum, mean, and minimum values in each direction, and repeated-measures ANOVA results.

Variables			Breathing Cycle	Non-Breathing Cycle	Breathing Cycle Following a Non-Breathing Cycle	F	ŋ^2^
			Mean ± SD [95%Cl]	Mean ± SD [95%Cl]	Mean ± SD [95%Cl]		
Hand	Max	X	1.39 ± 0.47	1.47 ± 0.26	1.38 ± 0.2	3.039	0.152
			[1.49; 1.29]	[1.54; 1.39]	[1.44; 1.31]		
		Y	2.54 ± 0.84	2.61 ± 0.61	2.54 ± 0.61	1.847	0.098
			[2.71; 2.36]	[2.79; 2.43]	[2.74; 2.34]		
		Z	1.64 ± 0.61	1.65 ± 0.47	1.7 ± 0.43	0.783	0.044
			[1.77; 1.52]	[1.79; 1.51]	[1.84; 1.56]		
	Mean	X	−0.01 ± 0.03	−0.01 ± 0.03	0 ± 0.02	0.861	0.048
			[−0.01; −0.02]	[0; −0.02]	[0; −0.01]		
		Y	0.93 ± 0.14	0.95 ± 0.13	0.93 ± 0.12	1.263	0.069
			[0.96; 0.90]	[0.98; 0.91]	[0.97; 0.89]		
		Z	0 ± 0.04	0 ± 0.04	0 ± 0.04	0.058	0.003
			[0.01; −0.01]	[0.01; −0.02]	[0.01; −0.01]		
	Min	X	−1.92; 0.34	−1.99; 0.36	−1.88; 0.31	2.973	0.149
			[−1.85; −1.99]	[−1.88; −2.09]	[−1.78; −1.98]		
		Y	−0.85 ± 0.25	−0.96 ± 0.27	−0.95 ± 0.23	0.698	0.039
			[−0.80; −0.90]	[−0.88; −1.04]	[−0.88; −1.03]		
		Z	−1.25 ± 0.25	−1.26 ± 0.25	−1.22 ± 0.16	0.928	0.052
			[−1.2; −1.31]	[−1.19; −1.33]	[−1.17; −1.27]		
Feet	Max	X	1.26 ± 0.25	1.36 ± 0.25	1.34 ± 0.21	0.612	0.035
			[1.31; 1.21]	[1.43; 1.28]	[1.41; 1.27]		
		Y	2.32 ± 0.29	2.36 ± 0.26	2.32 ± 0.26	3.772	0.180
			[2.38; 2.26]	[2.44; 2.28]	[2.41; 2.24]		
		Z	**0.77 ± 0.27 ^b^**	**0.98 ± 0.35 ^a^** **^,^** **^c^**	**0.81 ± 0.29 ^b^**	4.482	0.209
			[0.82; 0.71]	[1.08; 0.88]	[0.91; 0.72]		
	Mean	X	−0.01 ± 0.1	−0.01 ± 0.1	0.01 ± 0.07	1.127	0.062
			[0.01; −0.03]	[0.02; −0.04]	[0.03; −0.01]		
		Y	1.01 ± 0.20	0.98 ± 0.20	0.94 ± 0.18	3.046	0.152
			[1.05; 0.97]	[1.04; 0.92]	[1.00; 0.88]		
		Z	0.04 ± 0.1	**0.03 ± 0.09 ^c^**	**−0.01 ± 0.07 ^b^**	5.496	0.244
			[0.06; 0.02]	[0.06; 0.01]	[0.01; −0.04]		
	Min	X	−1.46 ± 0.33	−1.57 ± 0.39	−1.5 ± 0.28	0.934	0.052
			[−1.4; −1.53]	[−1.46; −1.68]	[−1.41; −1.59]		
		Y	−1.54 ± 0.61	−1.76 ± 0.53	−1.75 ± 0.54	1.696	0.091
			[−1.41; −1.66]	[−1.6; −1.91]	[−1.58; −1.93]		
		Z	−1.64 ± 0.47	−1.85 ± 0.54	−1.82 ± 0.38	3.613	0.175
			[−1.54; −1.73]	[−1.7; −2.01]	[−1.69; −1.95]		
Head	Max	X	0.12 ± 0.07	0.12 ± 0.08	0.13 ± 0.08	0.612	0.035
			[0.13; 0.1]	[0.15; 0.1]	[0.15; 0.1]		
		Y	1.27 ± 0.23	1.30 ± 0.17	1.31 ± 0.23	0.284	0.016
			[1.32; 1.23]	[1.35; 1.25]	[1.38; 1.23]		
		Z	1.17; 0.26	**1.01; 0.3 ^c^**	**1.20 ± 0.25 ^b^**	3.830	0.184
			[1.22; 1.12]	[1.1; 0.92]	[1.28; 1.12]		
	Mean	X	**0.02 ± 0.03 ^b^**	**0.02 ± 0.04 ^a^**	0.02 ± 0.04	3.726	0.180
			[0.02; 0.01]	[0.03; 0.01]	[0.03; 0.01]		
		Y	0.94 ± 0.14	**0.97 ± 0.13 ^c^**	**0.93 ± 0.13^b^**	4.101	0.194
			[0.97; 0.91]	[1.00; 0.93]	[0.97; 0.88]		
		Z	0.02 ± 0.06	−0.01 ± 0.09	0.02 ± 0.07	1.166	0.064
			[0.03; 0]	[0.01; −0.04]	[0.04; 0]		
	Min	X	−0.07 ± 0.06	−0.08 ± 0.05	−0.08 ± 0.04	3.083	0.154
			[−0.06; −0.09]	[−0.07; −0.1]	[−0.07; −0.09]		
		Y	0.58 ± 0.13	0.53 ± 0.17	0.53 ± 0.16	1.615	0.087
			[0.6; 0.55]	[0.58; 0.48]	[0.59; 0.48]		
		Z	−1.25 ± 0.39	**−1.06 ± 0.37 ^c^**	**−1.32 ± 0.33 ^b^**	3.507	0.171
			[−1.17; −1.33]	[−0.96; −1.17]	[−1.22; −1.43]		
CoM	Max	X	0.06 ± 0.04	0.07 ± 0.04	0.07 ± 0.04	1.290	0.071
			[0.07; 0.05]	[0.08; 0.05]	[0.08; 0.05]		
		Y	**1.24 ± 0.17 ^b^**	**1.30 ± 0.18 ^a,c^**	**1.24 ± 0.16 ^b^**	7.180	0.297
			[1.27; 1.21]	[1.35; 1.25]	[1.29; 1.18]		
		Z	0.30 ± 0.06	0.32 ± 0.07	0.32 ± 0.07	3.090	0.154
			[0.31; 0.29]	[0.34; 0.29]	[0.34; 0.3]		
	Mean	X	0.01 ± 0.04	0.01 ± 0.03	0.02 ± 0.04	3.124	0.155
			[0.02; 0.01]	[0.02; 0.01]	[0.03; 0.01]		
		Y	0.94 ± 0.14	0.96 ± 0.13	0.93 ± 0.13	2.459	0.126
			[0.97; 0.91]	[1.00; 0.92]	[0.97; 0.89]		
		Z	−0.01 ± 0.07	0 ± 0.03	0 ± 0.02	0.570	0.032
			[0.01; −0.02]	[0; −0.01]	[0; −0.01]		
	Min	X	**−0.02 ± 0.04 ^b^** **^,^** **^c^**	**−0.04 ± 0.04 ^a^**	**−0.04 ± 0.04 ^a^**	6.094	0.264
			[−0.02; −0.03]	[−0.02; −0.05]	[−0.02; −0.05]		
		Y	0.51 ± 0.12	0.50 ± 0.14	0.49 ± 0.13	0.884	0.049
			[0.54; 0.48]	[0.54; 0.46]	[0.54; 0.45]		
		Z	**−0.36 ± 0.07 ^c^**	−0.38 ± 0.09	**−0.43 ± 0.11 ^a^**	5.931	0.259
			[−0.35; −0.38]	[−0.35; −0.4]	[−0.39; −0.46]		

CoM—center of mass; v—velocity; ^a^—differs from the breathing cycle; ^b^—differs from the non-breathing cycle; ^c^—differs from the breathing cycle following a non-breathing cycle.

**Table 3:** Joint angles (hip, knee, and trunk) measured in relation to the sagittal plane throughout one cycle, and repeated-measures ANOVA results.

Variables	Breathing Cycle	Non-Breathing Cycle	Breathing Cycle Following a Non-Breathing Cycle	F	ŋ^2^
	Mean ± SD [95%Cl]	Mean ± SD [95%Cl]	Mean ± SD [95%Cl]		
Hip angle (°)	49.80 ± 8.70	50.90 ± 7.60	50.80 ± 7.50	0.852	0.048
	[51.60; 48.00]	[53.10; 48.70]	[53.30; 48.40]		
Knee angle (°)	137.00 ± 10.10	141.00 ± 11.90	141.10 ± 10.2	0.532	0.030
	[139.00; 134.90]	[144.50; 137.50]	[144.50; 137.80]		
Thorax angle (°)	44.10 ± 9.70	45.00 ± 13.30	47.10 ± 11.5	1.195	0.066
	[46.10; 42.10]	[48.90; 41.20]	[50.90; 43.30]		

**Table 4:** Intra-cycle mean velocity variations and repeated-measures ANOVA results.

Variables	Breathing Cycle	Non-Breathing Cycle	Breathing Cycle Following a Non-Breathing Cycle	F	ŋ^2^
	Mean ± SD [95%Cl]	Mean ± SD [95%Cl]	Mean ± SD [95%Cl]		
dv (%)	4.43 ± 6.77	3.74 ± 4.38	4.45 ± 5.51	0.275	0.052
	[7.86; 1.01]	[5.96; 1.53]	[7.57; 1.34]		
Fractal dimension	1.269 ± 0.095	1.291 ± 0.095	1.313 ± 0.079	0.891	0.050
	[1.288; 1.249]	[1.319; 1.264]	[1.339; 1.287]		

## Data Availability

Data are contained within the article.

## Appendix A

### Appendix A.1

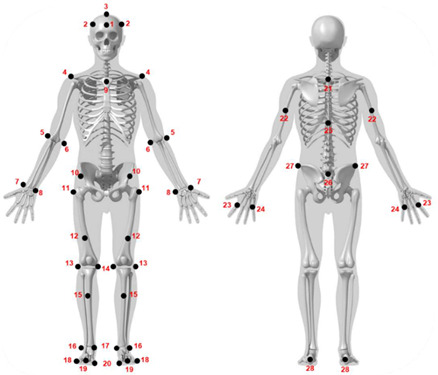

#	**Marker Name**	**Description**
1	HeadFront	Forehead, above the nose.
2	HeadL, HeadR	Just above the ear center.
3	HeadFront	On top of the head vertically above the ears.
4	LShoulderTop, RShoulderTop	On top of the shoulder (bony prominence).
5	LElbowOut, RElbowOut	On the outside of the elbow (bony prominence).
6	LElbowIn, RElbowIn	On the inside of the elbow (bony prominence).
7	LWristIn, RWristIn	On the inside of the wrist (bony prominence on the thumb side).
8	LWristOut, RWristOut	On the outside of the wrist (bony prominence on the pinky side).
9	Chest	Upper part of the sternum.
10	WaistLFront, WaistRFront	On the front of the pelvis (bony prominence).
11	LThighFrontHigh, RThighFrontHigh	Top of the thigh.
12	LThighFrontLow, RThighFrontLow	Above the kneecap.
13	LKneeOut, RKneeOut	On the outside of the knee (bony prominence).
14	LKneeIn, RKneeIn	On the inside of the knee (bony prominence).
15	LShinFrontHigh, RShinFrontHigh	Front of the shin.
16	LAnkleOut, RAnkleOut	On the outside of the ankle.
17	LAnkleIn, RAnkleIn	On the inside of the ankle.
18	LForefoot5, RForefoot5	Base of the toes on the outside.
19	LForefoot2, RForefoot2	Top of the foot tip.
20	LForefoot1, RForefoot1	Base of the toes on the inside.
21	SpineThoracic2	On the 2nd prominence below the biggest prominence on the top of the spine.
22	LArm, RArm	On the back of the upper arm.
23	LHand2, RHand2	Just below the index finger’s knuckle.
24	LHand5, RHand5	Just below the pinky finger’s knuckle.
25	SpineThoracic12	A few cm below the midpoint of the lower tip of the shoulder blades.
26	WaistBack	On the midpoint between the two prominences on the back of the pelvis.
27	WaistL, WaistR	On the sides of the pelvis (bony prominence).
28	LHeelBack, RHeelBack	Back of the heel.
Note: the prefixes “L” and “R” denote left and right sides.		

### Appendix A.2

Variables	Schematic Presentation
Hand variables	
1. **Vertical amplitude of the hand motor path during one upper limb’s full cycle.** Measured between the shallowest and deepest point of the hand motor path during one upper limb’s full cycle.	
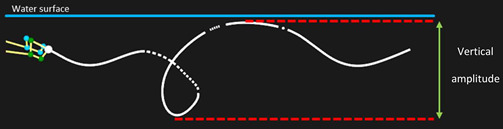
2. **Horizontal amplitude of the hand motor path during one upper limb’s full cycle.** Measured between the most rearward and forward point of the hand motor path during one upper limb’s full cycle.	
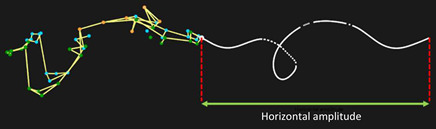
3. **Medio-lateral amplitude of the hand motor path during one upper limb’s full cycle.** Measured between the start of outsweep and start of insweep during one upper limb’s full cycle.	
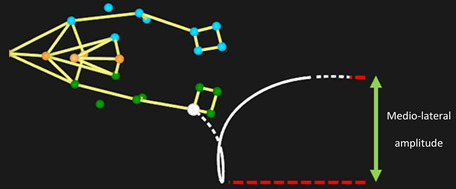
4. **Minimum depth of the hand motor path during one upper limb’s full cycle.** Measured between the water surface and the shallowest point of the hand motor path during one upper limb’s full cycle.	
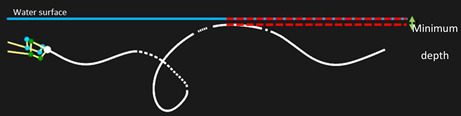
5. **Maximum depth of the hand motor path during one upper limb’s full cycle.** Measured between the water surface and the deepest point of the hand motor path during one upper limb’s full cycle.	
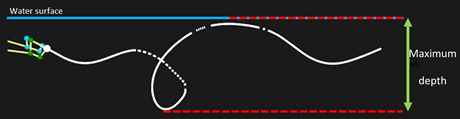
6. **Horizontality index of the hand motor path.** Ratio between the medio-lateral amplitude of hand motor path and the vertical amplitude of the hand motor path during the propulsive phase of one upper limb’s full cycle.	
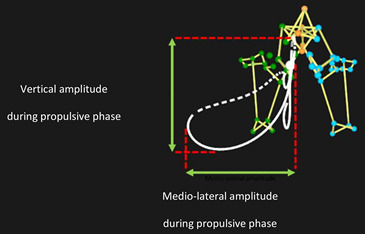
7. **Antero-posterior stabilization index of the hand motor path in the sagittal plane.** Ratio between the vertical amplitude of the hand motor path and the horizontal amplitude of the hand motor path during the propulsive phase of one upper limb’s full cycle.	
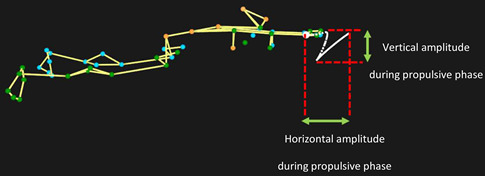
**Foot variables**	
8. **Vertical amplitude of the foot motor path during one full lower limb’s cycle.** Measured between the shallowest and deepest point of the foot motor path during one full lower limb’s cycle.	
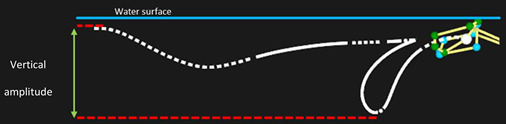
9. **Horizontal amplitude of the foot motor path during one full lower limb’s cycle.** Measured between the most rearward and forward point of the foot path during one full lower limb’s cycle.	
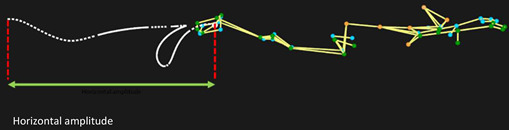
10. **Medio-lateral amplitude of the foot motor path during one full lower limb’s cycle.** Measured between the start of outsweep and the most lateral point of the motor path during one full lower limb’s cycle.	
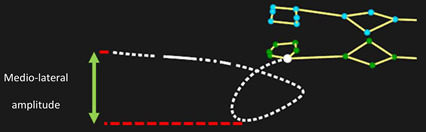
11. **Minimum depth of the foot motor path during one full lower limb’s cycle.** Measured between the water surface and the shallowest point of the foot motor path during one full lower limb’s cycle.	
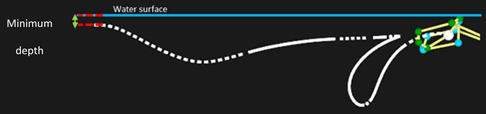
12. **Maximum depth of the foot motor path during one full lower limb’s cycle.** Measured between the water surface and the deepest point of the foot motor path during one full lower limbs cycle.	
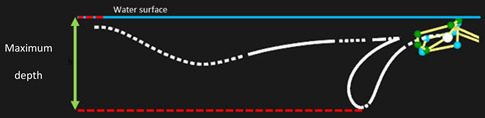
13. **Horizontality index of the foot motor path.** Ratio between the medio-lateral amplitude of foot motor path and the vertical amplitude of the foot motor path during the propulsive phase of one full lower limbs cycle.	
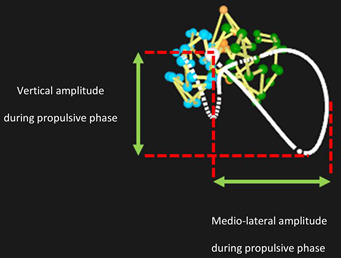
14. **Antero-posterior stabilization index of the foot motor path in the sagittal plane.** Ratio between the vertical amplitude of the foot motor path and the horizontal amplitude of the foot motor path during the propulsive phase of one full lower limbs cycle.	
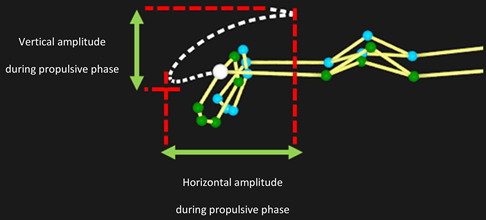
**Head variables**	
15. **Vertical amplitude of the head motor path during one full cycle.** Measured between the shallowest and deepest point of the head motor path during one full cycle.	
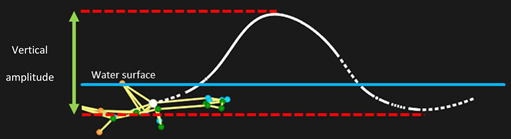
16. **Horizontal amplitude of the motor path of the head out of the water during one full cycle.** Measured between the rearmost and foremost points of the head out of the water during a complete cycle.	
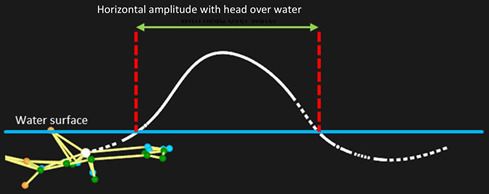
17. **Maximum depth of the head motor path during one full cycle.** Measured between the water surface and the deepest point of the head motor path during one full cycle.	
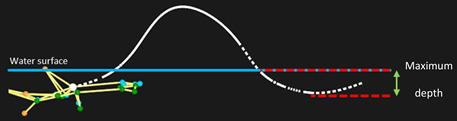
18. **Maximum elevation of the head motor path during one full cycle.** Measured between the water surface and the highest point above the water of the head motor path during one full cycle.	
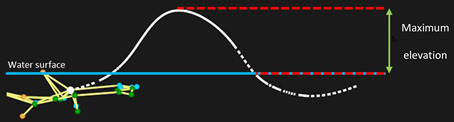
**Center of mass variables**	
19. **Vertical amplitude of the center of mass motor path during one full cycle.** Measured between the shallowest and deepest point of the center of mass motor path during one full cycle.	
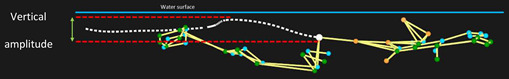
20. **Horizontal amplitude of the center of mass motor path during one full cycle.** Measured between the most rearward and forward point of the center of mass during one full cycle.	
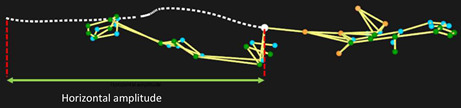
21. **Maximum depth of the center of mass motor path during one full cycle.** Measured between the water surface and the deepest point of the center of mass motor path during one full cycle.	
